# Grepafloxacin Versus Cefixime as Single-Dose Therapy for Uncomplicated Gonorrhea in Women

**DOI:** 10.1155/S1064744997000677

**Published:** 1997

**Authors:** T. F. Mroczkowski, E. W. Hook III, R. B. Jones, W. M. McCormack, D. H. Martin

**Affiliations:** 1 Department of Dermatology Tulane University School of Medicine 1340 Tulane Avenue New Orleans LA 70112 USA; 2 University of Alabama at Birmingham Jefferson County Health Department Birmingham AL USA; 3 Indiana University School of Medicine Bell Flower Clinic Indianapolis IN USA; 4 State University of New York Health Science Center at Brooklyn Kings County Hospital Center STD Clinic Brooklyn NY USA; 5 Department of Medicine Section of Infectious Diseases Louisiana State University New Orleans LA USA

## Abstract

*Objective: *To compare the efficacy and tolerance of single-dose grepafloxacin with cefixime for treatment of uncomplicated gonorrhea in women.

*Methods: *Women attending nine sexually-transmitted-disease clinics in the United States who had suspected uncomplicated gonorrhea were enrolled in an open study. Participants were randomized to receive single oral doses of grepafloxacin (400 mg) or cefixime (400 mg), and efficacy was evaluated in those who returned for follow-up assessment 5 to 10 days later. The primary measure of efficacy was microbiological response to therapy as determined by pre- and posttreatment culture results for *Neisseria gonorrhoeae*.

*Results: *Of 380 patients enrolled, 124 in the grepafloxacin group and 131 in the cefixime group were evaluated for microbiological response. Cervical gonococcal infections were eradicated in 99% of patients in both treatment groups, with only one persistent infection in each group. All pharyngeal (n = 15) and rectal (n = 32) gonococcal infections treated with grepafloxacin were cured, whereas 5 of 16 (31%) pharyngeal and 1 of 38 (3%) rectal infections failed to respond to cefixime. Although a third (123 of 386) of *N. gonorrhoeae* pretreatment isolates were resistant to penicillin or tetracycline, this had no impact on cure rates. Both drugs were well tolerated, with vaginitis being the most common treatment-related adverse event in each group.

*Conclusions: *This study shows that single-dose grepafloxacin is at least as effective as cefixime for treating women with uncomplicated cervical gonorrhea. Grepafloxacin also appears to be highly effective against extragenital infections.


Infectious Diseases in Obstetrics and Gynecology 5:370–375 (1997)
© 1998 Wiley-Liss, Inc.

### Grepafloxacin Versus Cefixime as Single-Dose Therapy for Uncomplicated Gonorrhea in Women

T.F. Mroczkowski,[1]* E.W. Hook III,[2] R.B. Jones,[3]
W.M. McCormack,[4] and D.H. Martin[5]

*[1]Tulane University School of Medicine, New Orleans, LA*
*[2]University of Alabama at Birmingham and Jefferson County Health Department, Birmingham, AL*
*[3]Indiana University School of Medicine and Bell Flower Clinic, Indianapolis, IN*
*[4]State University of New York Health Science Center at Brooklyn and Kings County Hospital Center STD Clinic, Brooklyn, NY*
*[5]Department of Medicine, Section of Infectious Diseases, Louisiana State University, New Orleans, LA*

### Abstract

***Objective:*** To compare the efficacy and tolerance of single-dose grepafloxacin with cefixime for treatment of uncomplicated gonorrhea in women.

***Methods:*** Women attending nine sexually-transmitted-disease clinics in the United States who had suspected uncomplicated gonorrhea were enrolled in an open study. Participants were randomized to receive single oral doses of grepafloxacin (400 mg) or cefixime (400 mg), and efficacy was evaluated in those who returned for follow-up assessment 5 to 10 days later. The primary measure of efficacy was microbiological response to therapy as determined by pre- and posttreatment culture results for *Neisseria gonorrhoeae.*

***Results:*** Of 380 patients enrolled, 124 in the grepafloxacin group and 131 in the cefixime group were evaluated for microbiological response. Cervical gonococcal infections were eradicated in 99% of patients in both treatment groups, with only one persistent infection in each group. All pharyngeal (n = 15) and rectal (n = 32) gonococcal infections treated with grepafloxacin were cured, whereas 5 of 16 (31%) pharyngeal and 1 of 38 (3%) rectal infections failed to respond to cefixime. Although a third (123 of 386) of *N. gonorrhoeae* pretreatment isolates were resistant to penicillin or tetracycline, this had no impact on cure rates. Both drugs were well tolerated, with vaginitis being the most common treatment-related adverse event in each group.

***Conclusions:*** This study shows that single-dose grepafloxacin is at least as effective as cefixime for treating women with uncomplicated cervical gonorrhea. Grepafloxacin also appears to be highly effective against extragenital infections. Infect. Dis. Obstet. Gynecol. 5:370–375, 1997.
© 1998 Wiley-Liss, Inc.

Key words
*Neisseria gonorrhoeae; Chlamydia trachomatis;* cefixime; grepafloxacin

Despite a decline of gonorrhea in the United States during the past two decades,[1] this sexually transmitted disease (STD) remains common in many inner cities, and some rural areas are currently experiencing high rates of infection.[2] Treatment of gonorrhea involved the almost exclusive use of penicillin and tetracyclines from the 1940s until the mid-1980s, when antibiotic resistance be-

*Correspondence to: Dr. Tomasz F. Mroczkowski, Department of Dermatology, Tulane University School of Medicine, 1340 Tulane Avenue, New Orleans, LA 70112.

Received 1 August 1997

Accepted 30 January 1998

came troublesome.[3] Today there are numerous antimicrobial agents at our disposal that are highly active against *Neisseria gonorrhoeae*, making the choice of treatment more difficult. All of the commercially available fluoroquinolones have proved effective as single-dose therapy of gonorrhea,[4, 5] including infections due to penicillinase-producing gonococci,[5] but only ciprofloxacin, ofloxacin, enoxacin, and norfloxacin are among the agents currently recommended by the Centers for Disease Control and Prevention.[6] After initial treatment for gonorrhea, follow-up therapy with an antibiotic regimen effective against possible coexistent infection with *Chlamydia trachomatis* is recommended.[6] Thus, the ideal antibiotic would be one that is effective against both gonococcal and chlamydial infections.

Grepafloxacin is a new, orally administered fluoroquinolone with excellent *in vitro* activity against *N. gonorrhoeae;* it is 4–8 fold more potent than ofloxacin against penicillin- and tetracycline-resistant strains[7] and more active than ofloxacin against *C. trachomatis.*[8] The drug is concentrated intracellularly,[9] has a long serum half-life (~12 hours) that allows for once-daily dosing,[10] and achieves high therapeutic levels in male and female genital tissue.[11, 12] These features indicate that grepafloxacin is a promising agent for treating common STDs.

A recent study has shown that a single 400-mg dose of grepafloxacin was as effective as the same dose of cefixime in the treatment of gonococcal urethritis in men.[13] The purpose of the present multicenter study was to investigate the efficacy and tolerance of grepafloxacin and cefixime in women with uncomplicated gonorrhea.

### SUBJECTS AND METHODS

The patients studied were recruited at nine public clinics for STDs in the United States between June 1993 and February 1995. The study sites were Druid STD Clinic, Baltimore, MD; Jefferson County Health Department STD Clinic, Birmingham, AL; Boston City Hospital STD Clinic, Boston, MA; Kings County Hospital Center STD Clinic, Brooklyn NY; Disease Control Service, Denver, CO; Bell Flower Clinic, Indianapolis, IN; Louisiana State University, Section of Infectious Diseases, Department of Medicine, New Orleans, LA; City and County of San Francisco, Department of Public Health, San Francisco, CA; and Harborview Medical Center STD Clinic, Seattle, WA. Women aged 16 years or older were eligible for enrolment if they had a gram-stained endocervical smear showing gram-negative diplococci within polymorphonuclear leukocytes, a recent untreated culture positive for *N. gonorrhoeae,* or sexual exposure to men with urethral gonorrhea within the two weeks prior to enrollment. All patients had to have positive cultures for *N. gonorrhoeae* at the initial visit to be evaluated.

Women of childbearing age were required to use a recognized contraceptive method during the study period. Patients were excluded if they had concomitant infection(s), such as complicated gonococcal infection, or disease that would compromise treatment evaluation; were pregnant or nursing; were known to have hypersensitivity to quinolone or cephalosporin antibiotics; had received antibiotics or any investigational drug within two weeks of enrollment; needed concurrent antimicrobial medication other than topical or antifungal agents; had received chronic treatment with warfarin, diflunisal, fluconazole, or theophylline; or had taken antacids or sucralfate within four hours of dosing. Patients with recent positive cultures for *C. trachomatis* were also excluded. The study was approved by the institutional review board at each participating center, and written informed consent was obtained from all patients before enrolment.

Specimens for gram staining of *N. gonorrhoeae* were obtained from the endocervix. Specimens for culture of *N. gonorrhoeae* were obtained from the cervix, pharynx, and rectum, and all patients had an endocervical culture for *C. trachomatis.* Blood and urine samples were taken for standard laboratory tests and syphilis serology. In addition, all participants had a urine pregnancy test immediately before enrolment.

Patients were randomly assigned to receive either a single oral dose of grepafloxacin (400 mg) or cefixime (400 mg) in an open study. The randomization schedule was based on a block design with four patient numbers (two grepafloxacin and two cefixime in each block). Each center assigned a specific regimen to each patient, enrolling them in the exact order specified on the list. Patients took the medication under supervision and were instructed to attend a follow-up visit 5 to 10 days later for repeat examination and cultures. Participants were advised to abstain from unprotected sexual intercourse during the study.

Specimens for *N. gonorrhoeae* isolation were inoculated directly onto Thayer-Martin selective agar and incubated for 24 to 48 hours at 36° C in a 5% $CO_2$ atmosphere. All presumptive gonococcal isolates were confirmed by standard methods[14] and stored at −70° C in Trypticase soy broth containing 15% glycerol prior to antibiotic susceptibility testing. The MICs for all isolates were determined by agar dilution.[15] Isolates were considered resistant to penicillin and tetracycline if MICs were ≥2.0 mg/L and resistant to cefixime if the MIC was >0.25 mg/L. Susceptibility to grepafloxacin was determined based on a tentative resistant breakpoint of >0.06 mg/L.[16] All isolates were tested for β-lactamase production by the chromogenic cephalosporin method.[17] Specimens for the isolation of *C. trachomatis* were inoculated into McCoy tissue culture cells and examined by indirect immunofluorescence.

The primary measure of study drug efficacy was microbiological response to therapy. Responses were classified as eradication or persistence on the basis of posttreatment cultures for *N. gonorrhoeae*. Overall tests of comparability between the two treatment groups were conducted for several categorical variables by using Cochran-Mantel-Haenszel statistics to adjust for the multicenter nature of the trial. Confidence intervals (CIs) of 95% for the differences between the two groups in microbiological response rates were calculated. A CI that crossed zero and remained within −10% or greater (for efficacy rates ≥ 90%) indicated treatment equivalence, whereas a lower limit >0% indicated grepafloxacin superiority.

### RESULTS

A total of 380 women, ranging in age from 15 to 54 years (mean age 23.5 years), were enrolled in the study (191 in the grepafloxacin group and 189 in the cefixime group), including one 15-year old who was enrolled by mistake. There were 332 black patients (87%), 28 Caucasian, and 20 of other racial origin. The patients enrolled in each treatment group were similar with respect to age, race, smoking habits, alcohol consumption, and illicit drug use. However, endocervical infection with *C. trachomatis* was more common among grepafloxacin-treated participants in that 31 of 191 women (16%) in the grepafloxacin group and 18 of 189 women (9.5%) in the cefixime group had *C. trachomatis* as determined by a positive culture at enrolment ($P$ = 0.066).

Of the 380 patients enrolled in the study, 86 had negative pretreatment cultures, 31 were lost to follow-up, five withdrew consent, and three were concomitantly using additional antibiotics. Thus, 255 patients had positive initial *N. gonorrhoeae* cultures and were available for microbiological evaluation at follow-up. Approximately one third of patients evaluated (80 of 255) with documented gonococcal cervicitis also had involvement of the rectum and/ or pharynx; the efficacy of both treatment regimens in eradicating *N. gonorrhoeae* from the various sites is summarized in Table 1.

TABLE 1. Eradication of *N. gonorrhoeae* by site of infection[a]

| | Isolates eradicated/isolates tested (%) | | |
|---|---|---|---|
| Site of infection | Grepafloxacin 400 mg | Cefixime 400 mg | *P*-value |
| Cervix | 114/115 (99.1)[b] | 124/125 (99.2)[b] | NS |
| Rectum | 32/32 (100) | 37/38 (97.4) | NS |
| Pharynx | 15/15 (100) | 11/16 (68.8) | 0.043 |
| Overall[c] | 123/124 (99.2) | 124/131 (94.7) | 0.067 |

[a]Defined as a negative culture four or more days posttreatment from bacteriologically evaluated patients. NS, not significant.
[b]Grepafloxacin equivalent to cefixime (95% CI, −9.75, 4.1%).
[c]Denotes eradication of *N. gonorrhoeae* from all anatomical sites; some patients had infection at more than one site.

Endocervical infections were eradicated in 99% of women in each treatment group. All extragenital infections treated with grepafloxacin (47 patients; 32 rectal and 15 pharyngeal infections) were cured, while 3% (1 of 38) of rectal infections and 31% (5 of 16) of pharyngeal infections did not respond to cefixime. When all infected sites are considered, 99% of patients given grepafloxacin were microbiologically cured, compared with 95% of those treated with cefixime. None of the eight patients with apparent treatment failure (one given grepafloxacin, seven given cefixime) said they had had unprotected sexual intercourse during the study period.

A total of 386 pretreatment isolates of *N. gonorrhoeae* were recovered from infected sites of 282 patients and tested for antibiotic susceptibility. Thirty-seven percent (70 of 191) of the isolates from grepafloxacin-treated patients and 27% (53 of 195) from cefixime-treated patients demonstrated

TABLE 2. Eradication of penicillin- and tetracycline-resistant isolates of *N. gonorrhoeae*[a]

| Site of infection | Isolate | Isolates eradicated/isolates tested (%) Grepafloxacin 400 mg | Cefixime 400 mg |
|---|---|---|---|
| Cervix | Penicillin-resistant | 26/27 (96.3) | 25/26 (96.2) |
| | Tetracycline-resistant | 19/21 (90.5) | 14/15 (93.3) |
| | β-lactamase-producing | 11/11 (100.0) | 8/8 (100.0) |
| Pharynx | Penicillin-resistant | 9/9 (100.0) | 0/1 (0.0) |
| | Tetracycline-resistant | 3/3 (100.0) | 0/1 (0.0) |
| | β-lactamase-producing | 4/4 (100.0) | — |
| Rectum | Penicillin-resistant | 3/4 (75.0) | 3/4 (75.0)[c] |
| | Tetracycline-resistant | 6/6 (100.0) | 5/6 (83.3)[c] |
| | β-lactamase-producing[b] | 1/1 (100.0) | 1/1 (100.0) |

[a]Includes all microbiologically evaluated patients with positive pretreatment cultures.
[b]β-lactamase-producing strains are a subset of penicillin-resistant strains and are included in the latter category.
[c]One cefixime-treated patient with rectal gonorrhea who was not treated successfully had a *Neisseria gonorrhoeae* isolate resistant to both penicillin and tetracycline.

resistance to either penicillin or tetracycline; 25 of 71 (35%) of penicillin-resistant gonococci produced β-lactamase. Both regimens were equally effective in eradicating penicillin- and tetracycline-resistant isolates from the cervix (Table 2). All 12 resistant isolates that caused pharyngeal infections in grepafloxacin recipients were eradicated, as were 9 of 10 rectal infections. However, cefixime failed to eradicate either of the two pharyngeal infections as well as two of the 10 rectal infections caused by resistant gonococci. All of the pretreatment isolates were initially susceptible to grepafloxacin (MIC ≤0.06 mg/L) and only one isolate, which was obtained from a patient randomized to grepafloxacin, had reduced susceptibility to cefixime (MIC > 0.25 mg/L).

Although not a primary goal of the study, an interesting finding was that a total of 49 patients had positive pretreatment cultures for *C. trachomatis*. Of these patients, 27 of 31 (87%) given grepafloxacin and 4 of 18 (22%) given cefixime had negative cultures for *C. trachomatis* at follow-up ($P < 0.001$). All patients with a confirmed *C. trachomatis* infection were treated with doxycycline. In all, about half (55%) of the patients in each treatment group who returned for follow-up examination were given a course of doxycycline to cover *C. trachomatis*. In some cases this was based on pretreatment culture results, but other clinics routinely treated all participants at follow-up. In addition, 13% of patients in each group received metronidazole for the treatment of trichomonal infection.

Adverse events that were considered treatment related occurred in 49 patients (62 events) in the grepafloxacin group and 28 patients (34 events) in the cefixime group ($P = 0.010$). All but nine of the events were mild to moderate and resolved spontaneously. One patient in each group experienced severe vaginal discharge (leukorrhea), which was considered to be drug related. One patient in the cefixime group was treated for a *C. trachomatis* infection, and another experienced headache and chest pain. In the grepafloxacin-treated group, one patient reported severe nausea and vomiting, and another had severe abdominal pain and diarrhea. The most frequently reported treatment-related events are presented in Table 3. Vaginitis was the most common individual treatment-related problem in each group, although when all gastrointestinal events were combined, they were more common overall. The overall incidence of treatment-related adverse events was higher in grepafloxacin-treated patients, but there was no significant difference in the incidences of individual adverse events between the treatment groups. In addition, none of the changes in laboratory values were considered clinically relevant.

### DISCUSSION

This study has shown that a single 400-mg oral dose of grepafloxacin is at least as effective as the same dose of cefixime for treatment of uncomplicated gonorrhea in women. Both drugs were highly effective (99% cure rate) in eradicating *N. gonorrhoeae* from the cervix, despite the fact that a third of the study patients were infected with penicillin- or tetracycline-resistant strains. Sixty-six of 70 (94%) of such isolates were eradicated by grepafloxacin, as were 47 of 53 (89%) by cefixime. All

TABLE 3. Summary of most common treatment-related adverse events[a]

| Adverse event | No. (%) of events reported after treatment: Grepafloxacin (N = 191) | Cefixime (N = 189) | P-value |
|---|---|---|---|
| Vaginitis | 12 (6.3) | 7 (3.7) | 0.347 |
| Nausea | 8 (4.2) | 4 (2.1) | 0.380 |
| Vomiting | 7 (3.7) | 2 (1.1) | 0.175 |
| Leukorrhea | 3 (1.6) | 5 (2.6) | 0.501 |
| Pruritis | 4 (2.1) | 2 (1.1) | 0.685 |
| Diarrhea | 4 (2.1) | 1 (0.5) | 0.372 |
| Taste alteration | 4 (2.1) | 0 | 0.123 |
| No. of patients with one or more treatment-related adverse event(s) | 49 (25.7) | 28 (14.8) | 0.010 |

[a]Events reported by more than 2% of patients in either treatment group.

pharyngeal and rectal gonococcal infections treated with grepafloxacin were microbiologically cured, while cefixime failed to resolve five (of 16) pharyngeal and one (of 38) rectal infections. Cefixime therefore appears to be unsatisfactory in eradicating *N. gonorrhoeae* from the pharynx. The 100% cure rate of extragenital infections seen with the use of grepafloxacin in this study was the same as that found in our previous study of grepafloxacin in the treatment of gonococcal urethritis in male patients.[13] Thus, grepafloxacin appears to be highly effective in both genital and extragenital gonococcal infections.

Both regimens were well tolerated, and the most common treatment-related adverse events were gastrointestinal in nature. Although treatment-related adverse events occurred with a higher frequency in the grepafloxacin group, no individual adverse event occurred with a significantly different frequency in either group.

Additional data was collected on the eradication of *C. trachomatis* in patients who were coinfected with this pathogen, although this was not a primary objective of the study. Like other cephalosporin antibiotics,[18] cefixime has little antichlamydial activity and, in this study, failed to cure most coexisting *C. trachomatis* endocervical infections. Grepafloxacin is active *in vitro* against *C. trachomatis*[8, 18] and was more effective than cefixime in eradicating this pathogen from patients who had positive cultures at pretreatment (87% vs. 22%, $P < 0.001$). This high rate of eradication is encouraging, but most *C. trachomatis* studies have a longer follow-up period, usually of four weeks. A single dose of grepafloxacin might, therefore, only be suppressive, and further follow-up of such cases may have revealed relapses. None of the present quinolones are effective as single-dose therapy for infections caused by *C. trachomatis,* and only ofloxacin has been approved for chlamydial urethritis and cervicitis.

As *C. trachomatis* is frequently present along with *N. gonorrhoeae* in genital infections, an agent that is effective in eliminating both pathogens would be a significant advance over conventional therapy using two or more antibiotics. Grepafloxacin has greater antigonococcal and antichlamydial activity *in vitro* than standard agents,[7, 8] and is two to 16 times more active than ceftriaxone and ofloxacin against susceptible, as well as penicillin- and tetracycline-resistant strains of *N. gonorrhoeae.*[7] Moreover, its activity is considerably greater than that of tetracycline against chlamydia, with some strains of *C. trachomatis* being up to 128 times more sensitive to grepafloxacin.[19] Such potency, together with a highly bactericidal action on *Chlamydia* species *in vitro,* may translate into good clinical efficacy.[18] These data, as well as our initial findings in nongonococcal urethritis and cervicitis clinical trials (in preparation), suggest that grepafloxacin may be useful as therapy for genital infections when both pathogens are present. Consequently, grepafloxacin is a promising new therapeutic agent for common STDs in both males and females.

### ACKNOWLEDGMENTS

The study was supported by Otsuka America Pharmaceutical, Inc.

### REFERENCES

1. Division of STD/HIV Prevention: Sexually transmitted disease surveillance, 1990. Atlanta: U.S. Department of Health and Human Services, Public Health Service, Centers for Prevention Services, 1991.
2. Thomas JC, Schoenbach VJ, Weiner DH, Parker EA, Earp JA: Rural gonorrhea in the Southeastern United States: a neglected epidemic? Am J Epidemiol 143:269–277, 1996.
3. Schwarcz SK, Zenilman JM, Schnell D, et al.: National surveillance of antimicrobial resistance in *Neisseria gonorrhoeae.* JAMA 264:1413–1417, 1990.
4. Moran JS, Levine WC: Drugs of choice for the treatment of uncomplicated gonococcal infections. Clin Infect Dis 20 (Suppl 1):S47–65, 1995.

5. Sivayathorn A: The use of fluoroquinolones in sexually transmitted diseases in South East Asia. Drugs 49 (Suppl 2):123–127, 1995.
6. Centers for Disease Control and Prevention: Sexually transmitted diseases treatment guidelines. MMWR 42 (Suppl 14):4–5, 1993.
7. Zenilman JM, Neumann T, Patton M, Reichart C: Antibacterial activities of OPC-17116, ofloxacin, and ciprofloxacin against 200 isolates of *Neisseria gonorrhoeae.* Antimicrob Agents Chemother 37:2244–2246, 1993.
8. Kimura M, Kishimoto T, Niki Y, Soejima R: In vitro and in vivo antichlamydial activities of newly developed quinolone antimicrobial agents. Antimicrob Agents Chemother 37:801–803, 1993.
9. Cook PJ, Andrews JM, Wise R, Honeybourne D, Moudgil H: Concentrations of OPC-17116, a new fluoroquinolone antibacterial, in serum and lung compartments. J Antimicrob Chemother 35:317–326, 1995.
10. Pitlick W, Hutson A, Lohse P, Malone D, Cabana J: The pharmacokinetics of OPC-17116, a new quinolone antibiotic, following multiple doses. In: Program and abstracts of the 31st Interscience Conference on Antimicrobial Agents and Chemotherapy. American Society for Microbiology, Washington DC. Abstract 1484, 1991.
11. Takahashi Y, Itoh Y, Doi T, Kuriyama M, Kawada Y: Penetration of OPC-17116, a new quinolone compound, into male genital tract tissue and its in vitro antibacterial activity. In: Program and abstracts of the 31st Interscience Conference on Antimicrobial Agents and Chemotherapy. American Society for Microbiology, Washington DC. Abstract 1486, 1991.
12. Matsuda S and the Japanese Collaborative Study Group of OPC-17116 in Gynaecology: Clinical experience with OPC-17116 in the treatment of gynaecological infections and its penetration into gynaecological tissues. Drugs 49 (Suppl 2):395–398, 1995.
13. Hook EW, McCormack WM, Martin D, et al.: Comparison of single-dose oral grepafloxacin with cefixime for treatment of uncomplicated gonorrhea in men. Antimicrob Agents Chemother. In press.
14. Morella JA, Jands WM, Doern GV: Neisseria and Branhamella. In: Balows A, Hausler WJ Jr, Herrman KL, Isenberg HD, Shadomy HJ (eds): Manual of Clinical Microbiology. 5th ed. Washington DC: American Society for Microbiology, pp 258–276, 1991.
15. National Committee for Clinical Laboratory Standards: Approved standard M7-A2. Methods for dilution antimicrobial susceptibility tests for bacteria that grow aerobically. 2nd ed. Villanova, PA: National Committee for Clinical Laboratory Standards, 1990.
16. Sewell DL, Barry AL, Allen SD, Fuchs PC, Murray PR, Tenover FC: Tentative interpretive criteria and quality control parameters for in-vitro susceptibility testing of *Neisseria gonorrhoeae* to two fluoroquinolones (PD 131628 and grepafloxacin, OPC-17116). J Antimicrob Chemother 37:139–143, 1996.
17. O'Callaghan CM, Morris A, Kirby SM, Shingler AM: Novel method for detection of beta-lactamase by using a chromogenic cephalosporin substrate. Antimicrob Agents Chemother 1:283–288, 1972.
18. Muytjens HL, Heessen FWA: In vitro activities of thirteen β-lactam antibiotics against *Chlamydia trachomatis.* Antimicrob Agents Chemother 22:520–521, 1982.
19. Wise R, Andrews JM, Brenwald N: The in-vitro activity of OPC-17116, a new 5-methyl substituted quinolone. J Antimicrob Chemother 31:497–504, 1993.
